# Morpholin-4-ium hydrogen tartrate

**DOI:** 10.1107/S1600536811055759

**Published:** 2012-01-07

**Authors:** Ming-Liang Liu

**Affiliations:** a Ordered Matter Science Research Center, Southeast University, Nanjing 211189, People’s Republic of China

## Abstract

In the title mol­ecular salt, C_4_H_10_NO^+^·C_4_H_5_O_6_^−^, the morpholinium cation adopts a chair conformation. The conformation of the C—C—C—C backbone of the monotartrate anion is close to *anti* [torsion angle = 173.18 (17)°], which is supported by two intra­molecular O—H⋯O hydrogen bonds. In the crystal, the components are linked by N—H—O and O—H—O hydrogen bonds, generating (001) sheets.

## Related literature

For a related structure, see: Ruble *et al.* (1976 ▶).
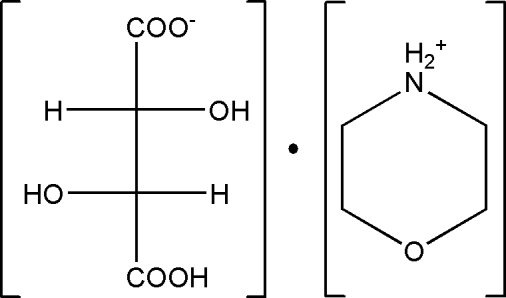


## Experimental

### 

#### Crystal data

C_4_H_10_NO^+^·C_4_H_5_O_6_^−^
*M* *_r_* = 237.21
Orthorhombic, 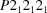

*a* = 7.2601 (15) Å
*b* = 9.1716 (18) Å
*c* = 16.283 (3) Å
*V* = 1084.2 (4) Å^3^
*Z* = 4
Mo *K*α radiation
μ = 0.13 mm^−1^
*T* = 293 K
0.36 × 0.32 × 0.28 mm

#### Data collection

Rigaku Mercury2 diffractometer
Absorption correction: multi-scan (*CrystalClear*; Rigaku, 2005 ▶) *T*_min_ = 0.954, *T*_max_ = 0.966
8960 measured reflections
1903 independent reflections
1747 reflections with *I* > 2σ(*I*)
*R*_int_ = 0.060

#### Refinement

*R*[*F* ^2^ > 2σ(*F* ^2^)] = 0.044
*wR*(*F* ^2^) = 0.097
*S* = 1.17
1903 reflections
146 parameters
H-atom parameters constrained
Δρ_max_ = 0.14 e Å^−3^
Δρ_min_ = −0.20 e Å^−3^

### 

Data collection: *CrystalClear* (Rigaku, 2005 ▶); cell refinement: *CrystalClear*; data reduction: *CrystalClear*; program(s) used to solve structure: *SHELXS97* (Sheldrick, 2008 ▶); program(s) used to refine structure: *SHELXL97* (Sheldrick, 2008 ▶); molecular graphics: *SHELXTL* (Sheldrick, 2008 ▶); software used to prepare material for publication: *SHELXTL*.

## Supplementary Material

Crystal structure: contains datablock(s) I, global. DOI: 10.1107/S1600536811055759/hb6576sup1.cif

Structure factors: contains datablock(s) I. DOI: 10.1107/S1600536811055759/hb6576Isup2.hkl

Additional supplementary materials:  crystallographic information; 3D view; checkCIF report

## Figures and Tables

**Table 1:** Hydrogen-bond geometry (Å, °)

*D*—H⋯*A*	*D*—H	H⋯*A*	*D*⋯*A*	*D*—H⋯*A*
N1—H1*A*⋯O2^i^	0.90	2.05	2.918 (3)	162
N1—H1*B*⋯O2^ii^	0.90	1.95	2.790 (3)	154
O1—H1⋯O2	0.82	2.09	2.600 (2)	120
O1—H1⋯O4^iii^	0.82	2.40	3.068 (2)	139
O5—H5⋯O3^iv^	0.82	1.73	2.529 (2)	165
O6—H6⋯O4	0.82	2.20	2.674 (2)	117
O6—H6⋯O1^v^	0.82	2.24	2.996 (2)	153

Symmetry codes: (i) 
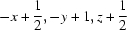
; (ii) 
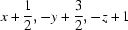
; (iii) 
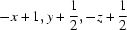
; (iv) 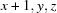; (v) 
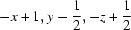
.

**Fig. 1.**
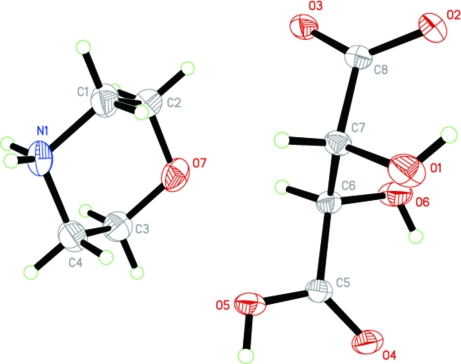
The molecular structure of the title compound, showing 30% probability displacement ellipsoids.

**Fig. 2.**
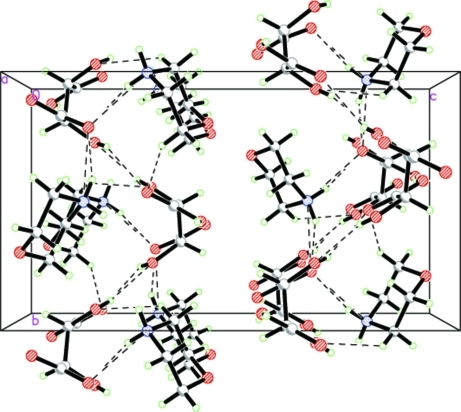
Crystal structure of the title compound with view along the b axis. Intermolecular interactions are shown as dashed lines.

## supplementary crystallographic information

### Experimental

0.87 g (0.01 mol) of morpholine was firstly dissolved in 30 ml of ethanol, to which 1.50 g (0.01 mol) of tartaric acid was added at ambient temperature. Colourless blocks were obtained by the slow evaporation of the above solution after 3 days in air.

The dielectric constant of the compound as a function of temperature indicates that the permittivity is basically temperature-independent (*ε* = C/(T–T_0_)), suggesting that this compound is not ferroelectric or there may be no distinct phase transition occurring within the measured temperature (below the melting point).

### Refinement

The absolute structure is indeterminate based on the present model. H atoms were placed in calculated positions (N—H = 0.89 Å; C—H = 0.93Å for C*sp^2^* atoms and C—H = 0.96Å and 0.97Å for C*sp^3^* atoms), assigned fixed *U*_iso_ values [*U*_iso_ = 1.2*U*eq(C*sp^2^*) and 1.5*U*eq(C*sp^3^*,*N*)] and allowed to ride.

### Crystal data

**Table:**

C_4_H_10_NO^+^·C_4_H_5_O_6_^−^	*F*(000) = 504
*M**_r_* = 237.21	*D*_x_ = 1.453 Mg m^−^^3^
Orthorhombic, *P*2_1_2_1_2_1_	Mo *K*α radiation, λ = 0.71073 Å
Hall symbol: P 2ac 2ab	Cell parameters from 1903 reflections
*a* = 7.2601 (15) Å	θ = 3.4–26.4°
*b* = 9.1716 (18) Å	µ = 0.13 mm^−^^1^
*c* = 16.283 (3) Å	*T* = 293 K
*V* = 1084.2 (4) Å^3^	Block, colourless
*Z* = 4	0.36 × 0.32 × 0.28 mm

### Data collection

**Table:**

Rigaku Mercury2 diffractometer	1747 reflections with *I* > \2s(*I*)
Radiation source: fine-focus sealed tube	*R*_int_ = 0.060
graphite	θ_max_ = 25°, θ_min_ = 3.1°
ω scans	*h* = −8→8
Absorption correction: multi-scan (*CrystalClear*; Rigaku, 2005)	*k* = −10→10
*T*_min_ = 0.954, *T*_max_ = 0.966	*l* = −19→19
8960 measured reflections	3 standard reflections every 180 reflections
1903 independent reflections	intensity decay: none

### Refinement

**Table:**

Refinement on *F*^2^	Primary atom site location: structure-invariant direct methods
Least-squares matrix: full	Secondary atom site location: difference Fourier map
*R*[*F*^2^ > 2σ(*F*^2^)] = 0.044	Hydrogen site location: inferred from neighbouring sites
*wR*(*F*^2^) = 0.097	H-atom parameters constrained
*S* = 1.17	*w* = 1/[σ^2^(*F*_o_^2^) + (0.0363*P*)^2^ + 0.0989*P*] where *P* = (*F*_o_^2^ + 2*F*_c_^2^)/3
1903 reflections	(Δ/σ)_max_ = 0.095
146 parameters	Δρ_max_ = 0.14 e Å^−^^3^
0 restraints	Δρ_min_ = −0.20 e Å^−^^3^

### Special details

**Table:**

Geometry. All esds (except the esd in the dihedral angle between two l.s. planes) are estimated using the full covariance matrix. The cell esds are taken into account individually in the estimation of esds in distances, angles and torsion angles; correlations between esds in cell parameters are only used when they are defined by crystal symmetry. An approximate (isotropic) treatment of cell esds is used for estimating esds involving l.s. planes.
Refinement. Refinement of F^2^ against ALL reflections. The weighted R-factor wR and goodness of fit S are based on F^2^, conventional R-factors R are based on F, with F set to zero for negative F^2^. The threshold expression of F^2^ > 2sigma(F^2^) is used only for calculating R-factors(gt) etc. and is not relevant to the choice of reflections for refinement. R-factors based on F^2^ are statistically about twice as large as those based on F, and R- factors based on ALL data will be even larger.

### Fractional atomic coordinates and isotropic or equivalent isotropic displacement parameters (Å^2^)

**Table:**

	*x*	*y*	*z*	*U*_iso_*/*U*_eq_	
O7	0.3787 (3)	0.3058 (2)	0.55071 (10)	0.0542 (5)	
N1	0.4758 (3)	0.4978 (2)	0.68097 (11)	0.0426 (5)	
H1A	0.4382	0.4480	0.7256	0.051*	
H1B	0.5249	0.5827	0.6979	0.051*	
C1	0.3161 (4)	0.5269 (3)	0.62661 (15)	0.0510 (7)	
H1C	0.3541	0.5882	0.5811	0.061*	
H1D	0.2210	0.5780	0.6570	0.061*	
C2	0.2413 (4)	0.3847 (3)	0.59447 (15)	0.0508 (7)	
H2A	0.1973	0.3262	0.6400	0.061*	
H2B	0.1378	0.4039	0.5584	0.061*	
C3	0.5307 (4)	0.2735 (3)	0.60333 (15)	0.0519 (7)	
H3A	0.6219	0.2178	0.5731	0.062*	
H3B	0.4885	0.2143	0.6490	0.062*	
C4	0.6178 (4)	0.4113 (3)	0.63583 (16)	0.0504 (7)	
H4A	0.7186	0.3871	0.6725	0.060*	
H4B	0.6668	0.4684	0.5907	0.060*	
O1	0.4383 (2)	0.75847 (19)	0.31549 (10)	0.0494 (5)	
H1	0.3531	0.7781	0.2843	0.074*	
O2	0.08530 (19)	0.71085 (17)	0.31349 (8)	0.0358 (4)	
O3	0.08075 (18)	0.60421 (18)	0.43779 (9)	0.0394 (4)	
O4	0.73405 (19)	0.46475 (19)	0.31136 (9)	0.0418 (4)	
O5	0.73379 (19)	0.58152 (19)	0.43315 (9)	0.0410 (4)	
H5	0.8459	0.5815	0.4269	0.062*	
O6	0.3670 (2)	0.44104 (19)	0.31002 (10)	0.0474 (5)	
H6	0.4483	0.4105	0.2796	0.071*	
C5	0.6557 (3)	0.5186 (2)	0.36993 (13)	0.0312 (5)	
C6	0.4477 (3)	0.5161 (3)	0.37717 (12)	0.0304 (5)	
H6A	0.4145	0.4652	0.4280	0.036*	
C7	0.3714 (3)	0.6722 (2)	0.38132 (13)	0.0304 (5)	
H7	0.4082	0.7168	0.4335	0.036*	
C8	0.1603 (3)	0.6624 (2)	0.37789 (13)	0.0284 (5)	

### Atomic displacement parameters (Å^2^)

**Table:**

	*U*^11^	*U*^22^	*U*^33^	*U*^12^	*U*^13^	*U*^23^
O7	0.0728 (12)	0.0500 (12)	0.0397 (9)	0.0035 (11)	−0.0084 (9)	−0.0083 (9)
N1	0.0568 (12)	0.0357 (12)	0.0351 (10)	−0.0088 (10)	0.0062 (9)	−0.0021 (9)
C1	0.0662 (17)	0.0440 (17)	0.0427 (13)	0.0129 (14)	0.0084 (12)	0.0050 (12)
C2	0.0513 (15)	0.0540 (18)	0.0472 (14)	0.0009 (14)	−0.0056 (12)	0.0016 (12)
C3	0.0590 (16)	0.0467 (18)	0.0499 (15)	0.0103 (14)	−0.0012 (13)	−0.0094 (13)
C4	0.0462 (15)	0.0577 (18)	0.0472 (14)	−0.0024 (14)	0.0061 (12)	−0.0044 (13)
O1	0.0317 (8)	0.0551 (12)	0.0614 (11)	−0.0088 (8)	0.0021 (8)	0.0244 (9)
O2	0.0332 (8)	0.0366 (9)	0.0377 (8)	0.0004 (7)	−0.0087 (7)	0.0026 (7)
O3	0.0229 (8)	0.0569 (11)	0.0384 (8)	−0.0009 (7)	0.0019 (7)	0.0068 (8)
O4	0.0298 (8)	0.0526 (11)	0.0429 (9)	0.0040 (8)	0.0065 (7)	−0.0073 (8)
O5	0.0211 (7)	0.0579 (11)	0.0441 (9)	0.0001 (7)	0.0015 (7)	−0.0070 (8)
O6	0.0299 (8)	0.0554 (11)	0.0568 (10)	0.0005 (8)	0.0002 (8)	−0.0227 (9)
C5	0.0257 (10)	0.0330 (13)	0.0349 (11)	0.0021 (10)	0.0013 (9)	0.0033 (10)
C6	0.0255 (10)	0.0337 (13)	0.0318 (11)	−0.0008 (10)	0.0023 (8)	−0.0002 (10)
C7	0.0244 (10)	0.0323 (13)	0.0344 (11)	−0.0025 (10)	−0.0003 (8)	0.0031 (10)
C8	0.0255 (10)	0.0245 (12)	0.0351 (11)	0.0000 (10)	−0.0015 (9)	−0.0034 (10)

### Geometric parameters (Å, °)

**Table:**

O7—C2	1.423 (3)	C4—H4B	0.9700
O7—C3	1.428 (3)	O1—C7	1.418 (2)
N1—C1	1.483 (3)	O1—H1	0.8203
N1—C4	1.494 (3)	O2—C8	1.263 (2)
N1—H1A	0.9006	O3—C8	1.253 (2)
N1—H1B	0.8996	O4—C5	1.216 (2)
C1—C2	1.507 (4)	O5—C5	1.309 (2)
C1—H1C	0.9700	O5—H5	0.8200
C1—H1D	0.9700	O6—C6	1.419 (2)
C2—H2A	0.9700	O6—H6	0.8198
C2—H2B	0.9700	C5—C6	1.515 (3)
C3—C4	1.510 (4)	C6—C7	1.536 (3)
C3—H3A	0.9700	C6—H6A	0.9800
C3—H3B	0.9700	C7—C8	1.536 (3)
C4—H4A	0.9700	C7—H7	0.9800
			
C2—O7—C3	110.29 (17)	N1—C4—H4A	109.9
C1—N1—C4	109.97 (18)	C3—C4—H4A	109.9
C1—N1—H1A	109.6	N1—C4—H4B	109.9
C4—N1—H1A	109.7	C3—C4—H4B	109.9
C1—N1—H1B	109.7	H4A—C4—H4B	108.3
C4—N1—H1B	109.7	C7—O1—H1	109.4
H1A—N1—H1B	108.2	C5—O5—H5	109.4
N1—C1—C2	109.5 (2)	C6—O6—H6	109.5
N1—C1—H1C	109.8	O4—C5—O5	126.37 (18)
C2—C1—H1C	109.8	O4—C5—C6	121.5 (2)
N1—C1—H1D	109.8	O5—C5—C6	112.16 (18)
C2—C1—H1D	109.8	O6—C6—C5	111.03 (17)
H1C—C1—H1D	108.2	O6—C6—C7	109.70 (17)
O7—C2—C1	111.2 (2)	C5—C6—C7	110.44 (18)
O7—C2—H2A	109.4	O6—C6—H6A	108.5
C1—C2—H2A	109.4	C5—C6—H6A	108.5
O7—C2—H2B	109.4	C7—C6—H6A	108.5
C1—C2—H2B	109.4	O1—C7—C6	111.28 (16)
H2A—C2—H2B	108.0	O1—C7—C8	110.32 (17)
O7—C3—C4	111.1 (2)	C6—C7—C8	107.67 (18)
O7—C3—H3A	109.4	O1—C7—H7	109.2
C4—C3—H3A	109.4	C6—C7—H7	109.2
O7—C3—H3B	109.4	C8—C7—H7	109.2
C4—C3—H3B	109.4	O3—C8—O2	126.69 (18)
H3A—C3—H3B	108.0	O3—C8—C7	117.18 (18)
N1—C4—C3	109.1 (2)	O2—C8—C7	116.10 (19)
			
C4—N1—C1—C2	56.0 (3)	O5—C5—C6—C7	61.0 (2)
C3—O7—C2—C1	60.4 (3)	O6—C6—C7—O1	−70.5 (2)
N1—C1—C2—O7	−58.4 (3)	C5—C6—C7—O1	52.2 (2)
C2—O7—C3—C4	−60.4 (3)	O6—C6—C7—C8	50.5 (2)
C1—N1—C4—C3	−55.9 (3)	C5—C6—C7—C8	173.18 (17)
O7—C3—C4—N1	58.0 (3)	O1—C7—C8—O3	−171.64 (18)
O4—C5—C6—O6	1.9 (3)	C6—C7—C8—O3	66.7 (2)
O5—C5—C6—O6	−177.08 (17)	O1—C7—C8—O2	10.1 (3)
O4—C5—C6—C7	−120.0 (2)	C6—C7—C8—O2	−111.5 (2)

### Hydrogen-bond geometry (Å, °)

**Table:**

*D*—H···*A*	*D*—H	H···*A*	*D*···*A*	*D*—H···*A*
N1—H1A···O2^i^	0.90	2.05	2.918 (3)	162
N1—H1B···O2^ii^	0.90	1.95	2.790 (3)	154
O1—H1···O2	0.82	2.09	2.600 (2)	120
O1—H1···O4^iii^	0.82	2.40	3.068 (2)	139
O5—H5···O3^iv^	0.82	1.73	2.529 (2)	165
O6—H6···O4	0.82	2.20	2.674 (2)	117
O6—H6···O1^v^	0.82	2.24	2.996 (2)	153

Symmetry codes: (i) −*x*+1/2, −*y*+1, *z*+1/2; (ii) *x*+1/2, −*y*+3/2, −*z*+1; (iii) −*x*+1, *y*+1/2, −*z*+1/2; (iv) *x*+1, *y*, *z*; (v) −*x*+1, *y*−1/2, −*z*+1/2.
